# Role of the Human Endogenous Retrovirus HERV-K18 in Autoimmune Disease Susceptibility: Study in the Spanish Population and Meta-Analysis

**DOI:** 10.1371/journal.pone.0062090

**Published:** 2013-04-25

**Authors:** Belén de la Hera, Jezabel Varadé, Marta García-Montojo, José Ramón Lamas, Ana de la Encarnación, Rafael Arroyo, Benjamín Fernández-Gutiérrez, Roberto Álvarez-Lafuente, Elena Urcelay

**Affiliations:** 1 Immunology Department, Hospital Clínico San Carlos, Instituto de Investigación Sanitaria del Hospital Clínico San Carlos (IdISSC), Madrid, Spain; 2 Multiple Sclerosis Unit, Neurology Department, Hospital Clínico San Carlos, Instituto de Investigación Sanitaria del Hospital Clínico San Carlos (IdISSC), Madrid, Spain; 3 Rheumatology Department, Hospital Clínico San Carlos, Instituto de Investigación Sanitaria del Hospital Clínico San Carlos (IdISSC), Madrid, Spain; National Institutes of Health, United States of America

## Abstract

**Background:**

Human endogenous retroviruses (HERVs) are genomic sequences that resulted from ancestral germ-line infections by exogenous retroviruses and therefore are transmitted in a Mendelian fashion. Increased HERV expression and antibodies to HERV antigens have been found in various autoimmune diseases. HERV-K18 in chromosome 1 was previously associated with type one diabetes and multiple sclerosis (MS). The etiology of these complex conditions has not been completely elucidated even after the powerful genome wide association studies (GWAS) performed. Nonetheless, this approach does not scrutinize the repetitive sequences within the genome, and part of the missing heritability could lie behind these sequences. We aimed at evaluating the role of HERV-K18 in chromosome 1 on autoimmune disease susceptibility.

**Methods:**

Two HERV-K18 SNPs (97Y/C and 154W/Stop substitutions) conforming three haplotypes were genotyped in Spanish cohorts of multiple sclerosis (n = 942), rheumatoid arthritis (n = 462) and ethnically matched controls (n = 601). Our findings were pooled in a meta-analysis including 5312 autoimmune patients and 4032 controls.

**Results:**

Significant associations of both HERV-K18 polymorphisms in chromosome 1 with MS patients stratified by HLA-DRB1*15∶01 were observed [97Y/C p = 0.02; OR (95% CI) = 1.5 (1.04–2.17) and 154W/Stop: p = 0.001; OR (95% CI) = 1.6 (1.19–2.16)]. Combined meta-analysis of the previously published association studies of HERV-K18 with different autoimmune diseases, together with data derived from Spanish cohorts, yielded a significant association of the HERV-K18.3 haplotype [97Y–154W: p_M-H_ = 0.0008; OR_M-H_ (95% CI) = 1.22 (1.09–1.38)].

**Conclusion:**

Association of the HERV-K18.3 haplotype in chromosome 1 with autoimmune-disease susceptibility was confirmed through meta-analysis.

## Introduction

Human endogenous retroviruses (HERVs) are sequences within the genome resulting from ancestral infections by exogenous retroviruses that were incorporated into germ-line DNA and therefore are inherited in a classical Mendelian pattern. They comprise approximately an 8% of the human genome [1] and have been detected in all vertebrates studied, including most apes, indicative of their evolutionary origin at least 25 million years ago. Different mechanisms have led to inactivation of most HERVs: recombination of their long terminal repeats with the excision of the proviral genome, hypermethylation of HERV promoters or mutations preventing the expression of functional HERV proteins. These mechanisms would support the concept that HERVs constitute “junk” DNA, but mounting evidence questions this view as some of them have physiological or pathological functions. The best example of their physiological role in the host is that of the HERV-W envelope protein known as syncytin, encoded on chromosome 7. This fusogenic protein expressed in the placenta is involved in the formation of the syncytiotrophoblast and decreased placental expression of this protein has been linked with pregnancy-induced hypertension [2]. An additional physiological role in spermatogenesis has been described for HERV-K in human males [3]. Moreover, the hypothesis that HERVs may be key factors in the pathogenesis of different diseases has been proposed. Apart from their specific expression reported in several cancer types, evidence of their pathogenic potential accumulates in a number of autoimmune diseases. Increased HERV expression from different families, as HERV-W, HERV-K or HERV-H, has been reported in rheumatoid arthritis (RA) [4], psoriasis [5], systemic lupus erythematosus [6] or multiple sclerosis (MS) [7]–[10]. Circulating antibodies to various HERV antigens have also been found in autoimmune patients [11]. Additionally, many reports show genetic association of different retrovirus with autoimmune diseases (i.e. association with MS of polymorphisms located near HERV-Fc or within a gen involved in retrovirus replication, *TRIM5* [12], [13]).

In this study we focused on the influence of a copy of the HERV-K family, located in transcriptional antisense within the first intron of the *CD48* gene in chromosome 1q23.3. In 1997, Conrad and collaborators [14] found that the *env* gene of a new HERV-K endogenous retrovirus encoded a superantigen in insulin-dependent diabetes. Later, it was suggested that the tissue tropism of an exogenous virus might trigger the organ specific HERV-K18 superantigen response, leading to the expansion of autoreactive T cells [15]. Elevated HERV-K18 superantigen was also found in juvenile rheumatoid arthritis [16]. The HERV-K18 in chromosome 1 is an insert of 9235 bp with three variants and all of them encode superantigens [17]. Nonetheless, HERV-K18 expression might be a consequence rather than the cause of autoimmune conditions.

The search for the aetiology of these complex diseases has undergone a spectacular advance in recent times with the identification through genome wide association studies (GWAS) of literally hundreds of common single nucleotide polymorphisms (SNPs) involved in their susceptibility. However, although GWAS have provided valuable insights in the underlying causes of complex traits, they have explained relatively little of their heritability and the issue of where the “missing heritability” lies is a matter of debate [18]. There are several possible explanations as to the missing genetic basis. One of them points to the identified marker SNPs as imperfect proxies for the actual causal mutations that led to the association, which frequency could be low and would remain undetected. However, recent studies claim that rare variants in predisposition genes discovered by GWAS are not likely to make a large contribution to inherited risk in complex diseases [19]. Furthermore, gene-environment interactions may be relevant to fully elucidate the causes of these complex diseases. Another possible explanation is that repetitive sequences in the genome have not been adequately scrutinized through GWAS. With these considerations in mind, in the present work we aimed at studying the three haplotypes of HERV-K18 on chromosome 1 previously described associated with diabetes and MS risk [20], [21] and at testing their influence on autoimmune disease susceptibility, provided the shared genetic susceptibility evidenced among these autoimmune conditions [22]. Additionally, increased HERV-K18 transcription was reported in cultured synoviocytes derived from biopsies of RA patients compared to controls [23]. A meta-analysis has been conducted including data from MS and RA Spanish cohorts combined with previously published results. In both traits, human herpesvirus 6 (HHV-6) or Epstein–Barr virus (EBV) infections have been often times outlined as environmental triggers of the diseases [24], [25]. Moreover, HHV-6 and EBV induce transcriptional activation of the endogenous superantigen HERV-K18, independently of virus replication [26], [27].

## Materials and Methods

### Ethics Statement

The study was approved by the Ethics Committee of the Hospital Clínico San Carlos, Madrid, Spain. Patients and ethnically matched controls gave written informed consent.

### Meta-analysis

**Figure 1:**
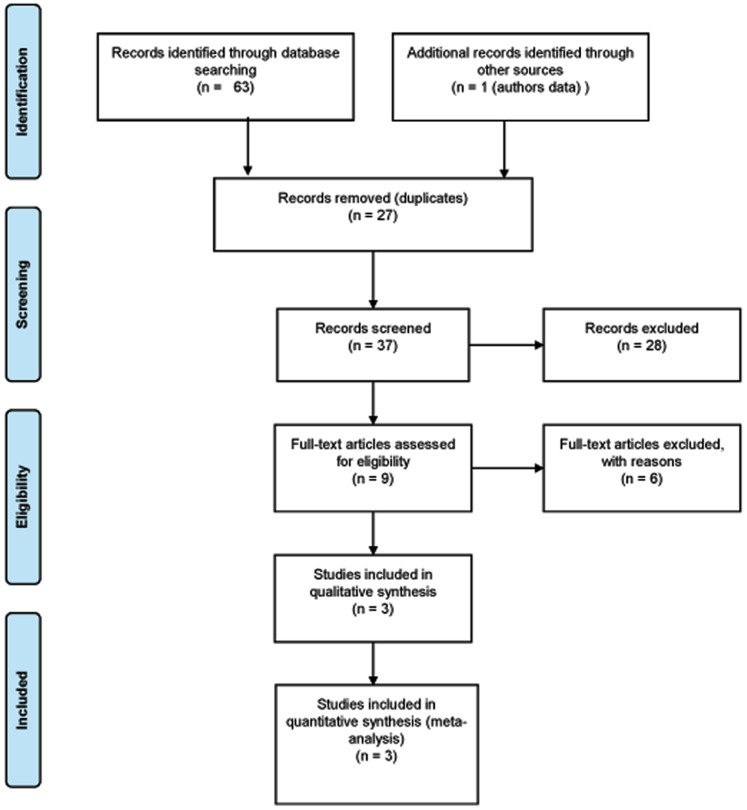
Strategy used for the selection of studies finally included in the meta-analysis.

We performed a comprehensive search strategy of various electronic databases (MEDLINE (1966 - December 2012), Cochrane Database of Systematic Reviews (1991- December 2012) and EMBASE (1980- December 2012) by combining the terms: “HERVk-18”, “IDDMK(1,2)22” and “Human endogenous retrovirus-k18”. Additionally, a manual search of all references was conducted among the identified studies and relevant review articles (See Figure 1 and Checklist S1). This search rendered 37 articles published to date and all the association studies involved the copy on chromosome 1. Neither date nor language restrictions were imposed. The association studies considered for further analysis were required to hold information about *HERV-K18* haplotypes. We also included non published data from Hospital Clinico San Carlos (HCSC) cohort.

### Patients

The Spanish case-control study included 942 MS, 462 RA patients and 601 ethnically matched controls consecutively recruited from a single centre (Hospital Clínico San Carlos, Madrid, Spain).

MS Spanish patients (63% female) were diagnosed based on the McDonald criteria [28] and 36% of them carried the human leukocyte antigen HLA DRB1*1501 risk allele. Most patients suffered relapsing remitting MS (79%), 11% of them showed the secondary progressive clinical form and 9% of them were classified as primary progressive patients. Their mean age at MS onset was 29±9.

RA Spanish patients (74% female) were diagnosed according to American College of Rheumatology criteria [29]. The mean age of the RA patients at the time of disease onset was 51±15, 56% of patients were shared epitope (SE) positive and 52% were anti-cyclic citrullinated peptide (anti-CCP) antibody positive. SE identification was achieved by using Lifecodes HLA-DRB Typing Kit (Tepnel Diagnostics Ltd., Abingdon, Oxon, UK).

### Genotyping

DNA was extracted from peripheral blood by a standard salting out method. A fragment of 6417 bp of HERV-K18 *env* (accession number AF333069, GRCh37.p10 (160666969–160673386); www.ensembl.org, October-2012) was amplified by polymerase chain reaction (PCR) [Cycle conditions: initial denaturation: 94°C/5 min, 35 cycles: 94°C/30 sec, 62°C/30 sec, 68°C/5 min, and final extension of 68°C/10 min] using a primer located in the proviral sequence and the other mapping to the *CD48* sequence (5′-CCCACTCTAATGCAAGCTC-3′ and 5′-CATGGGAAATAGGGAAGCTG-3′). Within this amplicon, two SNPs, a Y/C substitution at amino acid position 97 (SNP1) and another W/Stop at position 154 (SNP2), discriminate the three haplotypes previously described (AF134984, Y18890, and AF333069) [20], [21]. They were analyzed by Taqman technology using 384 well plates in a 7900HT Fast Real-Time PCR system, under the conditions recommended by the manufacturer (Applied Biosystems, Foster City, CA, USA). Genotyping success was over 95% for all groups of patients and controls. No departure from Hardy Weinberg equilibrium was observed in the control group.

### Statistical Analysis

Statistical analyses were performed with standard software (SPSS v15 and Review Manager RevMan v. 5.0.).

Haplotypic frequencies were estimated using the expectation-maximization and partition-ligation algorithms implemented in the Haploview software [30].

For the meta-analysis, Odds ratios (ORs) and 95% confidence intervals (CIs) were calculated by using raw data for each study and for the pooled population. The Der Simonian and Laird random effects model was used according to the results of the tests of heterogeneity. The combined effect for heterogeneity was calculated by taking the estimated inverse variance. The effect of each study was weighted for its number of patients. P value <0.10 defines a significant degree of heterogeneity and we also used the I^2^ statistic, with a cut-off point of 25%, to assess heterogeneity between the studies. A sensitivity analysis was performed to test the relative influence of each study on the results. Studies were sequentially dropped, and the effect on the change in the overall degree of heterogeneity was determined.

## Results

**Figure 2:**
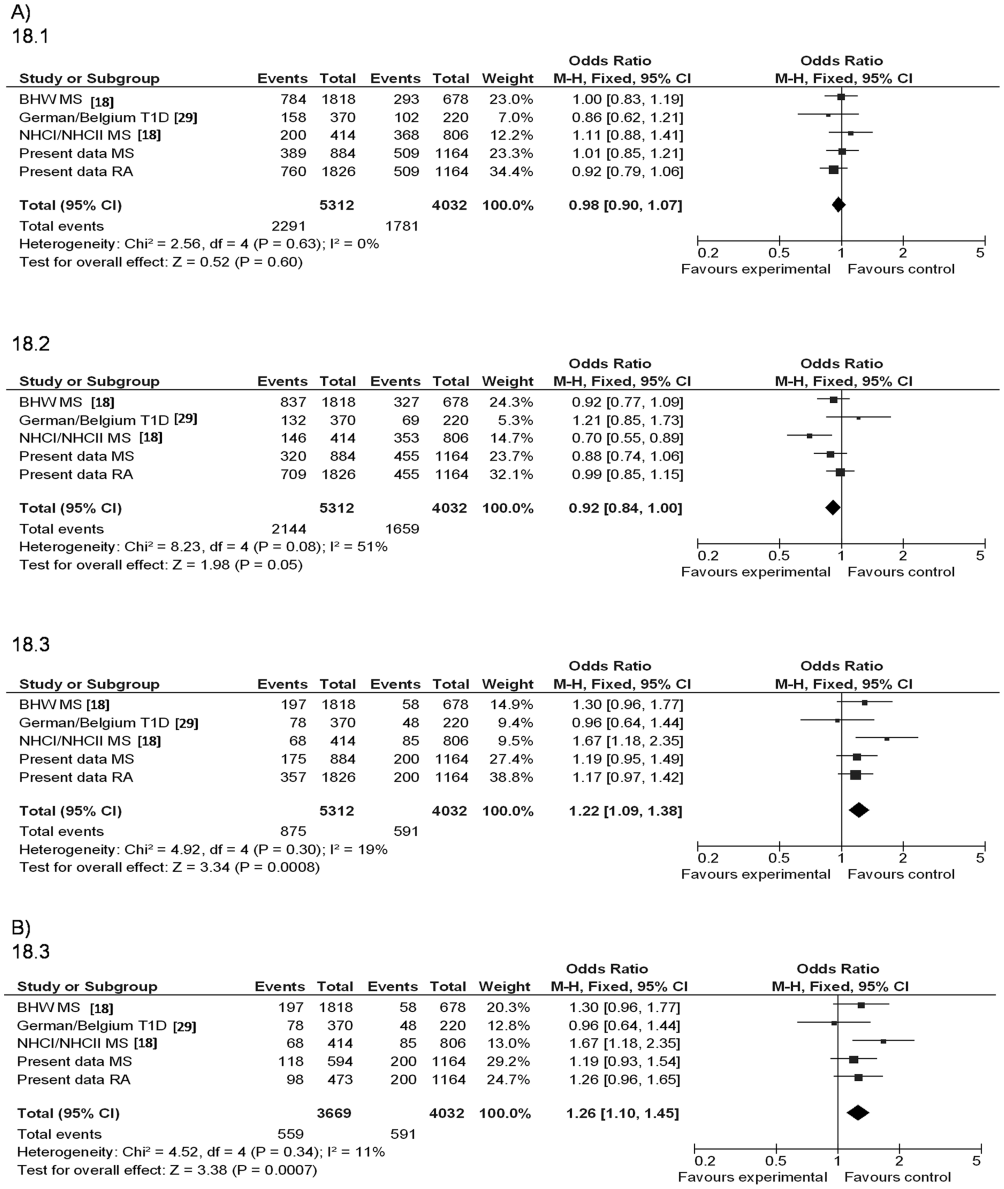
Role of HERV-K18 haplotypes A) Meta-analysis of case-control studies in different autoimmune disease cohorts; B) Spanish data stratified by HLA susceptibility alleles (DRB1*15∶01 in MS and shared epitope in RA).

In order to ascertain the real impact of the HERV-K18 haplotypes in chromosome 1 on autoimmune disease risk, we performed a meta-analysis with 5312 patients and 4032 controls. After the systematic review, only 4 studies remained for further analysis [20], [21], [31], [32]. Due to remarkable differences in allele frequencies found between Japanese and European populations, data from a non-European ancestry study [32] were not included in the meta-analysis. Other Transmission Disequilibrium Test (TDT) study lacking case-control data [20] was also discarded. Finally the meta-analysis pooled data from the previously published cohorts [21], [31] and our MS and RA Spanish cohorts (Figure 2).

In the Spanish samples, a 6417 bp fragment of chromosome 1q22.3 was amplified by PCR using a primer located in the HERV-K18 *env* proviral sequence and the other mapping to the *CD48* sequence (as described in Methods). Case-control studies from the pre-amplified fragment of the Spanish samples were conducted with the two SNPs covering the haplotypic diversity of the HERV-K18 chromosomal region: a change Y/C at amino acid position 97 and another change W/Stop at 154. As shown in Fig. 2A, no association was observed for haplotype 18.1 (97Y-154Stop; SNP1*A-SNP2*A: p = 0.60, OR = 0.98) or for haplotype 18.2 after performing sensitivity analyses and eliminating the source of heterogeneity (97C-154W; SNP1*G-SNP2*G: p = 0.31, OR = 0.95). Haplotype 18.3 evidenced an overall effect [97Y-154W; SNP1*A-SNP2*G: p = 0.0008; OR (95% CI) = 1.22 (1.09–1.38)] with low heterogeneity among data of the reported cohorts and ours (I^2^ = 19%). When the RA and MS Spanish cohorts were stratified by the HLA risk factor (Fig. 2B), the shared epitope alleles and HLA-DRB1*15∶01 respectively, even higher homogeneity among cohorts was found (I^2^ = 11%).

The genotypic frequencies for SNP1 and SNP2 in the Spanish patients and controls are summarized in Table 1. Significant differences were observed for both SNPs when MS patients were stratified by the well-known susceptibility factor HLA-DRB1*15∶01 (MS 1501+ vs. Controls: GG vs. AG+AA: p = 0.02, OR = 1.50 and p = 0.001, OR = 1.60, for SNP1 and SNP2 respectively). No significant association of the tested SNPs with RA risk was reached in the Spanish cohort, most probably due to statistical power restrictions.

**Table 1:** Genotypic frequencies of polymorphisms within the HERV-K18 sequence in autoimmune disease Spanish patients and controls.

				SNP1			SNP2		
				AA	AG	GG	GG	AG	AA
				n (%)	n (%)	n (%)	n (%)	n (%)	n (%)
**Controls**				225 (37)	286 (48)	90 (15)	188 (31)	298 (50)	112 (19)
**MS**	**Overall**			372 (39)	417 (44)	153 (16)	334 (36)	422 (45)	175 (19)
	**1501**		**1501^+^**	114 (37)	128 (42)	64 (21)^1^	128 (42)^2^	121 (40)	53 (18)
			**1501^−^**	196 (39)	241 (48)	70 (14)	155 (31)	246 (49)	102 (20)
**RA**	**Overall**			197 (43)	192 (42)	65 (14)	132 (30)	231 (52)	79 (18)
	**SE**		**SE^+^**	107 (44)	105 (43)	31 (13)	65 (28)	133 (56)	38 (16)
			**SE^−^**	64 (40)	69 (43)	27 (17)	51 (32)	77 (49)	29 (18)

1 GG vs AG+AA: MS 1501^+^ vs 1501^−^: p = 0.01; MS 1501^+^ vs. Controls: p = 0.02, OR (95%CI) = 1.50 (1.04–2.17).

2 GG vs AG+AA: MS 1501^+^ vs 1501^−^: p = 0.0009; MS 1501^+^ vs. Controls: p = 0.001, OR (95%CI) = 1.60 (1.19–2.16).

## Discussion

Autoimmune diseases are multifactorial conditions determined by the interplay of environmental and genetic components. Particularly, viral infections have been proposed to trigger these diseases in the background of genetic predisposition. In both, multiple sclerosis and rheumatoid arthritis, the main genetic contribution was long ago attributed to specific human leukocyte antigen (HLA) class II alleles on the short arm of chromosome 6p21. Other genes increasing risk to both diseases have been recently ascertained through GWAS [33], [34], and a shared genetic background among them evidenced. However, the genetic component has been incompletely disentangled maybe due to partial coverage of the genome. In fact, associated genes which initially passed undetected by GWAS were sometimes identified through other experimental approaches. One limitation of whole genome association studies corresponds to the difficulty to unambiguously identify repetitive elements by the standard hybridization-based methods used, and consequently polymorphisms in HERVs would remain undetected in GWAS platforms. Prior amplification of genomic target regions, as the one performed in the present work, is required to successfully characterize endogenous retroviral sequences.

Our aim in the present work was the analysis of the role of the HERV-K18 haplotypes in chromosome 1 on autoimmune disease susceptibility. A search with the HERV-K18 *env* sequence (accession number AF333069, GRCh37.p10 (160666969–160673386); www.ensembl.org, October-2012) evidenced 332 positions in the genome showing total or partial homology, and 27 out of them presented over 85% homology and over 80% of the total length of HERV-K18 *env*. Among those 27 copies, 21 have open reading frames (ORFs) with one of both of the studied SNPs. No SNP previously found associated with any autoimmune disease through GWAS was observed in the 200 Kb- sequence surrounding those 27 copies. As mentioned above, we focused on the association previously observed within chromosome 1. Former studies regarding the association of HERV-K18 with other autoimmune disease, type 1 diabetes, reported apparently inconsistent results [20], [31], [32]. The genetic evidence presented here corroborates the HERV-K18 influence on autoimmune disease predisposition. Our data is fully concordant with a published report showing the involvement of HERV-K18 on the genetic risk to MS [21]. Provided that HERV-K18 superantigens are strictly dependent on Major Histocompatibility Complex class II for T cell activation, genetic epistasis between both loci is plausible and it seems from our data that a stronger association is found in the subgroup of MS patients carrying the HLA-DRB1*15∶01 risk allele (Table 1).

Given the location of HERV-K18 within the intron of the *CD48* gene, the etiology of a putative *CD48* variant in linkage disequilibrium with the 18.3 haplotype found associated could not be disregarded. In terms of epigenetic regulation, the ENCODE project data show the signal of the H3K4ME1 histone in the promoter region of *CD48* in an immortalized cell line of B lymphocytes, GM12873, indicative of a DNAse hypersensitive site [35]. The impact of CD48 on T cell activation has been recognized with the description of severe T cell signaling defects in the CD48-knockout mouse [36]. T cells reorganize their surface molecules to form a well-structured contact zone with antigen presenting cells, known as immunological synapse, and CD48 in particular was firmly demonstrated to amplify T-cell receptor signaling [37]. Moreover, it has been recently reported that retrotransposable elements, including endogenous retroviruses, can act as an evolutionary mechanism for coordinately boosting the expression of many genes, suggesting that they become functional promoters [38].

The present results reside at the crossroads of environmental and genetic factors influencing autoimmune disease predisposition. Recently, mechanistic data about the transactivation of HERV-K18 by EBV have been released [39], [40], and our work supports the role of the HERV-K18 haplotype on chromosome 1 in autoimmune pathogenesis. In summary, we report the association of the HERV-K18 18.3 haplotype on chromosome 1 with autoimmune disease susceptibility and further studies are warranted to fully elucidate the specific mechanism underlying this association.

## Supporting Information

Checklist S1 **Reported items for meta-analysis.**
(DOCX)
